# ClpP participates in stress tolerance, biofilm formation, antimicrobial tolerance, and virulence of *Enterococcus faecalis*

**DOI:** 10.1186/s12866-020-1719-9

**Published:** 2020-02-07

**Authors:** Jinxin Zheng, Yang Wu, Zhiwei Lin, Guangfu Wang, Sibo Jiang, Xiang Sun, Haopeng Tu, Zhijian Yu, Di Qu

**Affiliations:** 1 Department of Infectious Diseases and the Key Lab of Endogenous Infection, Shenzhen Nanshan People’s Hospital and The 6th Affiliated Hospital of Shenzhen University Health Science Center, Shenzhen, 518052 China; 2 Key Laboratory of Medical Molecular Virology of Ministries of Education and Health, School of Basic Medical Science and Institutes of Biomedical Sciences, Shanghai Medical College of Fudan University, Shanghai, 200032 China; 3 Department of Pharmaceutics, University of Florida, Orlando, 32827 USA

**Keywords:** *Enterococcus faecalis*, ClpP, Stress tolerance, Biofilm formation, Virulence, Antimicrobial tolerance

## Abstract

**Background:**

ClpP is important for bacterial growth and plays an indispensable role in cellular protein quality control systems by refolding or degrading damaged proteins, but the physiological significance of ClpP in *Enterococcus faecalis* remains obscure. A *clpP* deletion mutant (△*clpP*) was constructed using the *E. faecalis* OG1RF strain to clarify the effect of ClpP on *E. faecalis.* The global abundance of proteins was determined by a mass spectrometer with tandem mass tag labeling.

**Results:**

The Δ*clpP* mutant strain showed impaired growth at 20 °C or 45 °C at 5% NaCl or 2 mM H_2_O_2_. The number of surviving Δ*clpP* mutants decreased after exposure to the high concentration (50× minimal inhibitory concentration) of linezolid or minocycline for 96 h. The Δ*clpP* mutant strain also demonstrated decreased biofilm formation but increased virulence in a *Galleria mellonella* model. The mass spectrometry proteomics data indicated that the abundances of 135 proteins changed (111 increased, 24 decreased) in the Δ*clpP* mutant strain. Among those, the abundances of stress response or virulence relating proteins: FsrA response regulator, gelatinase GelE, regulatory protein Spx (*spxA*), heat-inducible transcription repressor HrcA, transcriptional regulator CtsR, ATPase/chaperone ClpC, acetyl esterase/lipase, and chaperonin GroEL increased in the Δ*clpP* mutant strain; however, the abundances of ribosomal protein L4/L1 family protein (*rplD*), ribosomal protein L7/L12 (*rplL2*), 50S ribosomal protein L13 (*rplM*), L18 (*rplR*), L20 (*rplT*), 30S ribosomal protein S14 (*rpsN2*) and S18 (*rpsR*) all decreased. The abundances of biofilm formation-related adapter protein MecA increased, while the abundances of dihydroorotase (*pyrC*), orotate phosphoribosyltransferase (*pyrE*), and orotidine-5′-phosphate decarboxylase (*pyrF*) all decreased in the Δ*clpP* mutant strain.

**Conclusion:**

The present study demonstrates that ClpP participates in stress tolerance, biofilm formation, antimicrobial tolerance, and virulence of *E. faecalis.*

## Background

*Enterococcus faecalis* has emerged as a significant cause of nosocomial infections in the last two decades, resulting in urinary tract infections, bacteremia, prosthetic joint infection, abdominal-pelvic infections, and endocarditis [1]. *E. faecalis* has resistance to many commonly used antimicrobial agents, and vancomycin-resistant enterococci (VRE) has emerged as a major cause of nosocomial infection outbreaks in recent years [2]. In addition to drug resistance, *E. faecalis* carries a high capacity for biofilm formation; more than 40% of clinical *E. faecalis* isolates can form biofilms [3–7]. Several virulence factors have been associated with *E. faecalis* biofilm formation. For example, the enterococcal surface protein (*esp*) was found to adhere to and colonize abiotic surfaces that participate in *E. faecalis* biofilm formation, and gelatinase (*gelE*) that can hydrolyze gelatin, collagen, and hemoglobin was also implicated in the adherence and biofilm formation of *E. faecalis* [6, 8–10]. However, *esp* and *gelE* were found to have no association with biofilm formation in other extensive collections of *E. faecalis* isolates [11–13]. Thus, the genes involved in the *E. faecalis* biofilm formation remain controversial and obscure. Other unknown factors may also participate in this important process.

The Hsp100/Clp family protein ClpP is important for bacterial growth and plays an indispensable role in cellular protein quality control systems by refolding or degrading damaged proteins in stressed cells [14]. ClpP was also associated with biofilm formation in some pathogenic species. For example, the biofilms of *Streptococcus mutans*, *Staphylococcus epidermidis*, *Pseudomonas aeruginosa*, and *Actinobacillus pleuropneumoniae* decreased when *clpP* was mutated [15–18]. However, the capacities to form biofilms were enhanced when *clpP* was mutated in *Staphylococcus aureus, Haemophilus parasuis*, and *Porphyromonas gingivalis* [19–21]. The roles of *clpP* in bacterial biofilm formation are not been fully understood. RNA levels of *clpP* of *S. epidermidis* were decreased by the *agr* quorum-sensing system, but in *S. aureus* Newman and USA300 strains, *agrA* and *agrC* RNA levels were significantly reduced in *clpP* mutants [16, 21]. *clpP* affected the expression of the transcriptional regulators *csrA* and *rpoD* and a possible biofilm repressor *luxS* to enhance *H. parasuis* biofilm formation, and it negatively adjusted the surface exposure of the minor fimbrial (Mfa) protein that promotes the biofilm formation of *P. gingivalis* [19, 20]. The role of *clpP* on *E. faecalis* biofilm formation remains unknown to date.

In addition to bacterial growth, stress response, and biofilm formation, ClpP also influences the virulence and antibacterial tolerance of several pathogenic organisms. *clpP* mutation significantly attenuated *Streptococcus pneumoniae* virulence in a murine intraperitoneal infection model. Expression of the virulence-related pneumolysin and pneumococcal antigen were dependent on the ClpP protease [22]. Michel found that the abundance of the *agr* system and *agr*-dependent extracellular virulence factors were diminished in the *S. aureus* 8325 △*clpP* strain [23]. In *Legionella pneumophila*, the *clpP*-deficient mutant strain was unable to escape the endosome-lysosomal pathway in host cells [24]. The *clpP* deletion mutation also attenuated *Salmonella* Typhimurium virulence through dysregulation of RpoS and indirect control of CsrA and the SPI genes [25]. In *S. aureus*, in addition to the stress response, biofilm formation, and virulence, the truncating mutation in *clpP* is responsible for the raised vancomycin resistance in VISA strain LR5P1-V3 [26]. Bæk found that inactivation of the components of the ClpXP protease substantially increased β-lactam resistance in the *S. aureus* USA300 strain, while the *clpP* mutant strain displayed significantly thicker cell walls, increased peptidoglycan cross-linking, and altered composition of monomeric muropeptide species compared to wild type [27]. As mentioned above, *E. faecalis* shows resistance to many antimicrobial agents; however, whether the *clpP* is involved in *E. faecalis* resistance to antimicrobials, especially vancomycin (VRE), is still unclear.

To obtain a more comprehensive understanding of the role of ClpP protease in the *E. faecalis* stress response, biofilm formation, virulence, and antimicrobial tolerance, a △*clpP* strain was constructed in *E. faecalis* strain OG1RF. The global abundance of proteins was detected with an Orbitrap Q Exactive HF-X mass spectrometer with tandem mass tag (TMT) labeling.

## Results

### Construction of the *clpP* deletion mutant and complemented strain

To explore the role of ClpP in *E. faecalis*, we constructed a *clpP* deletion mutant in the *E. faecalis* OG1RF strain using the temperature-sensitive plasmid pJRS233. The deletion mutant strain was verified by polymerase chain reaction (PCR) and direct sequencing and was termed the OG1RF Δ*clpP* mutant strain. The complemented Δ*clpP* strain (Δ*clpP*/pIB166::*clpP*) was constructed using shuttle vector pIB166 and also verified by PCR and direct sequencing. The Δ*clpP* strain containing the empty vector pIB166 was designated as OG1RF Δ*clpP*/pIB166. *clpP* RNA levels of all the above four *E. faecalis* OG1RF strains were determined by quantitative reverse transcription PCR (RT-qPCR) as shown in Additional file 1: Figure S1.

### Δ*clpP* mutant strain showed impaired growth at 20 °C, 45 °C, 5%NaCl, or 2 mM H_2_O_2_

**Fig. 1:**
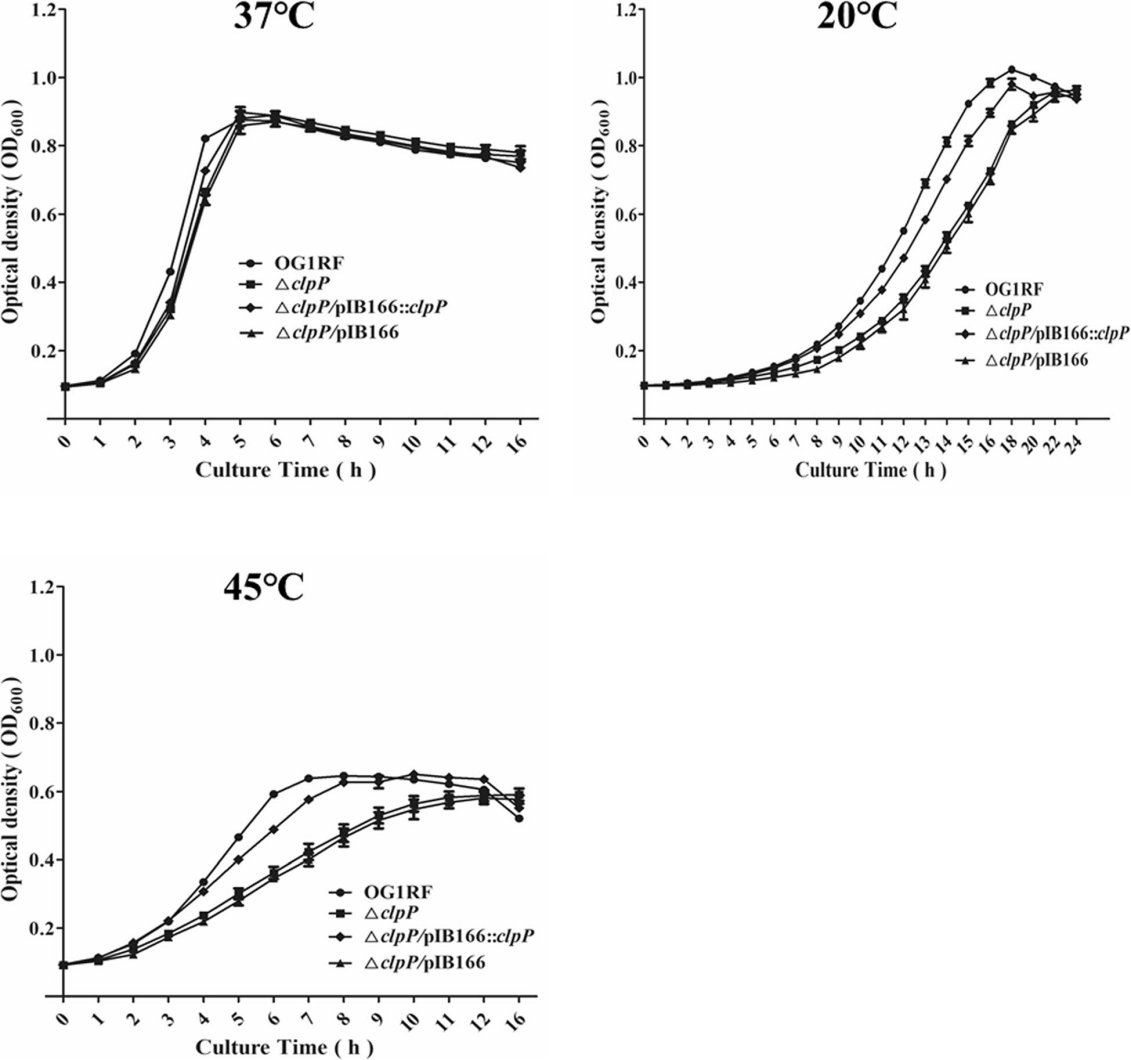
Effect of *clpP* deletion on *E. faecalis* growth at 37 °C, 20 °C, and 45 °C. Three independent experiments were performed, and the data represent means ± SD

**Fig. 2:**
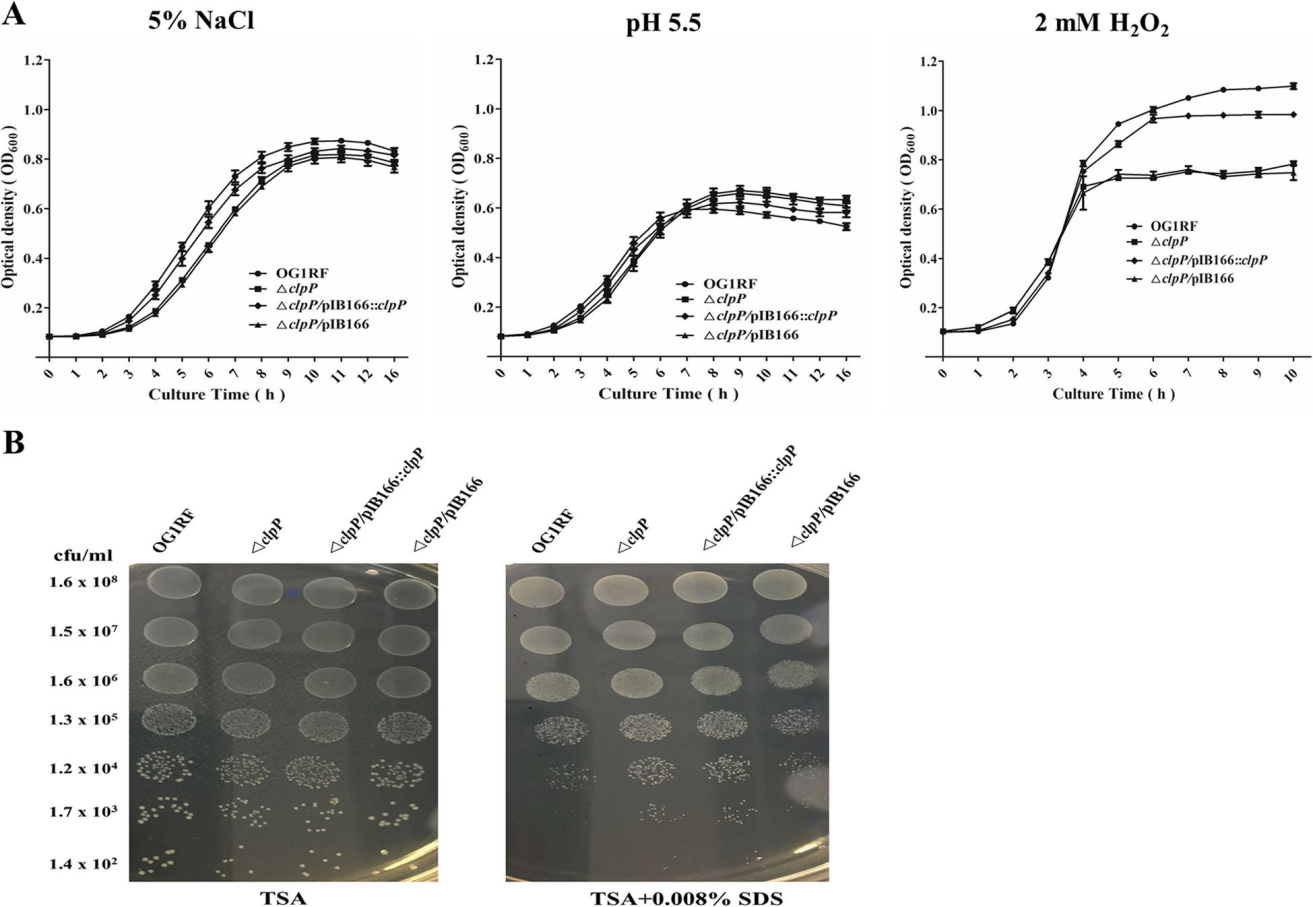
Sensitivity of the Δ*clpP* mutant to hyperosmotic pressure, low pH, oxidative stress, and SDS. **a** Overnight cultures of the *E. faecalis* strains were diluted in TSB containing 5% NaCl or with pH 5.5 and then incubated at 37 °C for 16 h, or in TSB containing 2 mM H_2_O_2_ incubated at 37 °C for 10 h. Three independent experiments were performed, and the data represent means ± SD. **b** The *E. faecalis* strains were spotted onto TSB agar plates containing 0.008% SDS and incubated for 24 h at 37 °C. Three independent experiments were performed, and the representative results are shown

Previous research indicated that ClpP participated in the *S. aureus* stress response to low or high temperature and the oxidative stress response [23]; however, these issues have not been studied in *E. faecalis*. Thus, we first investigated the effects of *clpP* deletion on *E. faecalis* growth under the stresses of low or high temperature, hyperosmotic pressure, low pH, and oxidative stress. At 37 °C, there were no significant growth differences between the *E. faecalis* OG1RF parent strain and its Δ*clpP* mutant. However, under 20 °C or 45 °C, the Δ*clpP* mutant strain showed a lower optical density at 600 nm (OD_600_) than was observed for the wild-type strain after entering logarithmic phase growth (Fig. 1). As shown in Fig. 2, Δ*clpP* mutant strain growth was also impaired under 5% NaCl (logarithmic phase) or 2 mM H_2_O_2_ (later logarithmic phase or stationary phase).

### *clpP* deletion leads to decreased biofilm formation

**Fig. 3:**
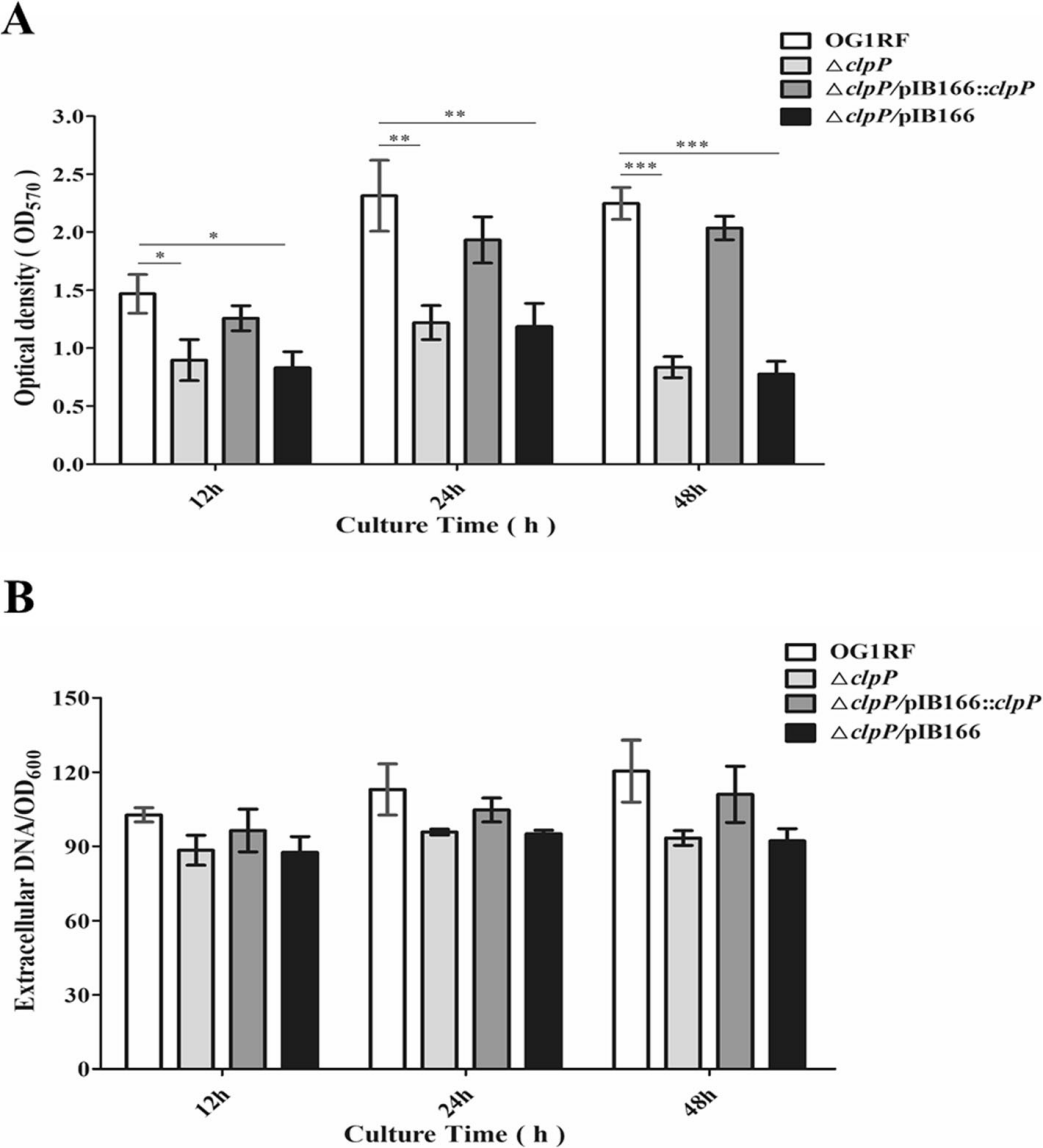
Effects of the Δ*clpP* mutant on *E. faecalis* biofilm formation and eDNA release. **a** The biofilms of *E. faecalis* strains were stained with crystal violet, and OD_570_ values were measured. *P < 0.05, **P < 0.01, ***P < 0.001 (Student’s t test). **b** PI-bound eDNA of *E. faecalis* strains was measured by a Varioskan™ LUX multimode microplate reader. Three independent experiments were performed, and the data represent means ± SD

Polystyrene microtiter plate assays were performed to evaluate the role of *clpP* in the biofilm formation of *E. faecalis* under static conditions. The biofilm formation of *E. faecalis* OG1RF parent strain and its Δ*clpP* mutant was monitored at 12, 24, and 48 h on microtiter plates stained with crystal violet (CV), and OD_570_ values were determined. The biofilms of the Δ*clpP* mutant strain (OD_570_, 0.835 ± 0.091) were significantly decreased compared with that of the parent strain (OD_570_, 2.247 ± 0.138, *P* < 0.001, Student’s t test) after incubation for 48 h, and this outcome was also observed after incubation for 12 or 24 h (Fig. 3 a). We further investigated extracellular DNA (eDNA) release during *E. faecalis* biofilm formation but found no differences between the Δ*clpP* mutant and its parent strain (Fig. 3 b).

### Antimicrobial tolerance of the Δ*clpP* mutant strain

**Fig. 4:**
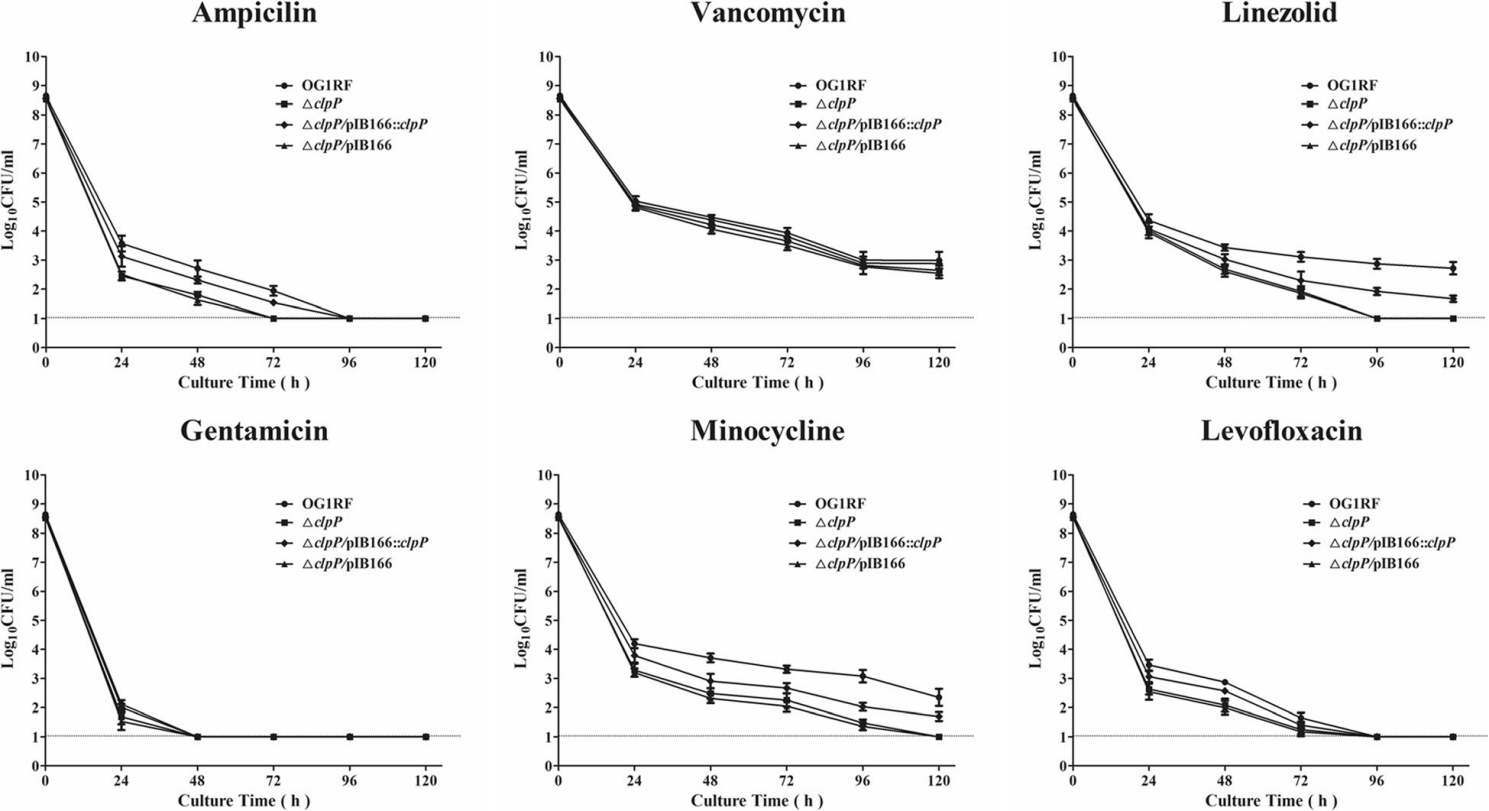
Survival of the Δ*clpP* mutant and the parent strain with antimicrobial exposure over time. Three independent experiments were performed, and the data represent means ± SD. The dashed line indicates the assay’s detection limit

The minimal inhibitory concentrations (MICs) of eight antimicrobials for *E. faecalis* were detected by the broth microdilution method, and the MICs for the Δ*clpP* mutant strain were similar to those of the parent strain (Additional file 4: Table S1). To determine which antimicrobial concentrations ensured that only drug-tolerant bacterial cells survived, we performed time-killing assays for six antimicrobials. Based on previous research [28] and our preliminary results, the concentrations of six antimicrobials were set at 50× MIC. As shown in Fig. 4, the surviving bacteria of the Δ*clpP* mutant strain (log_10_ colony-forming units [CFU]/mL, under the detection limit) were significantly decreased compared with those of the parent strain (log_10_CFU/mL, 2.873 ± 0.243, *P* < 0.001, Student’s t test) after 96-h exposure to linezolid. After 96-h exposure to minocycline, the surviving bacteria of the Δ*clpP* mutant strain (log_10_CFU/mL, 1.477 ± 0.171) were also decreased compared with the parent strain (log_10_CFU/mL, 3.078 ± 0.303, *P* < 0.01, Student’s t test).

### Δ*clpP* mutant leads to increased *E. faecalis* virulence

**Fig. 5:**
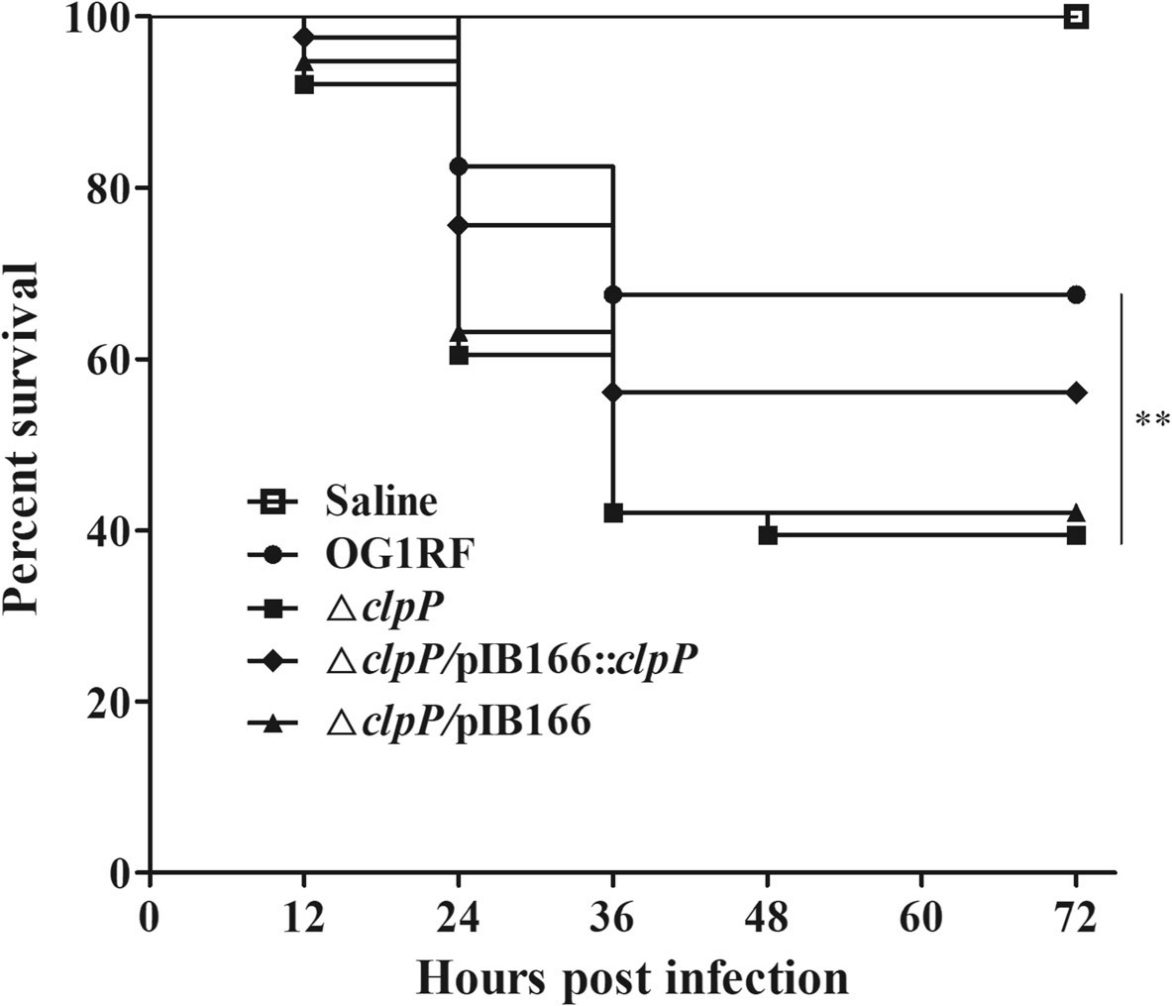
Deletion of *clpP* leads to increased virulence of *E. faecalis*. *G. mellonella* were infected with 20 μL inocula of *E. faecalis* strains containing 5 × 10^6^ CFU/mL, and the survival of *G. mellonella* larvae was recorded at 12-h intervals for 72 h p.i. Data were collected from three independent experiments, and representative results are shown. **P < 0.01 (log-rank test)

The virulence of *E. faecalis* strains was detected by the infection of *Galleria mellonella* larvae. The survival of *G. mellonella* larvae infected with the Δ*clpP* mutant strain (15/40, 37.5%) significantly decreased compared with the parent strain (28/40, 70.0%, *P* < 0.01, log-rank test) at 72 h post infection (p.i.) (Fig. 5). The complemented △*clpP/*pIB166::*clpP* strain (23/40, 57.5%) showed a partially restored survival ability.

### Comparison of the global protein abundances of the Δ*clpP* mutant and parent strain

We compared the global protein abundances of the Δ*clpP* mutant and parent strain. The total proteins were extracted from logarithmic phase (4 h) and stationary phase (12 h) bacteria, and their abundances were determined on an Orbitrap Q Exactive HF-X mass spectrometer with TMT labeling. The protein quantitation results were statistically analyzed by Mann-Whitney tests, and the significant ratios, defined as *P* < 0.05 and ratio > 1.2 or < 0.83 (fold change, FC), were used to screen differential abundance proteins (DAPs). The protein quantitation results are given as the means from two independent experiments, and the repeatability of the two independent experiments was evaluated by the coefficient of variation (CV). As shown in Additional file 2: Figure S2, the CV for the two independent experiments was very low. All DAPs are summarized in Table 1. The abundances of 135 proteins changed in the Δ*clpP* mutant strain, of which 111 increased and 24 decreased.

**Table 1:** Global differential abundance of proteins between the Δ*clpP* mutant and its parent strains

Protein ID (locus_tag, gene name)	Description or predicted function	Protein abundance ratio of △*clpP*/WT	
		4 h	12 h
Abundance-increased			
AEA95010.1 (*OG1RF_12323*)	hypothetical protein	2.953	2.642
AEA94213.1 (*OG1RF_11526*, *gelE*)	gelatinase GelE	–	2.577
AEA93457.1 (*OG1RF_10770*)	aspartate 4-decarboxylase	–	1.964
AEA95218.1 (*OG1RF_12531*, *ctsR*)	transcriptional regulator CtsR	2.165	1.894
AEA93816.1 (OG1RF_11129)	hypothetical protein	2.086	–
AEA95217.1 (*OG1RF_12530, clpC*)	ATPase/chaperone ClpC, probable specificity factor for ClpP protease	1.906	1.802
AEA94588.1 (*OG1RF_11901*)	HD domain protein	–	1.905
AEA94933.1(*OG1RF_12246*)	Acetyl esterase/lipase	–	1.873
AEA94972.1 (*OG1RF_12285, cbh*)	putative penicillin amidase	–	1.790
AEA94928.1 (*OG1RF_12241*)	LysR family transcriptional regulator	1.799	–
AEA93656.1 (*OG1RF_10969*)	beta-lactamase	–	1.770
AEA92985.1 (*OG1RF_10298, mtlD*)	mannitol-1-phosphate 5-dehydrogenase	1.711	–
AEA93763.1(*OG1RF_11076, hrcA*)	heat-inducible transcription repressor HrcA	–	1.692
AEA93514.1 (*OG1RF_10827*)	hypothetical protein	1.655	–
AEA94757.1 (*OG1RF_12070*)	hypothetical protein	1.651	–
AEA95193.1 (*OG1RF_12506*)	cell wall surface anchor family protein	–	1.609
AEA95024.1 (*OG1RF_12337*)	Hydrolase	1.585	2.182
AEA94737.1 (*OG1RF_12050*)	HAD-superfamily hydrolase	–	1.606
AEA94733.1 (*OG1RF_12046, mecA*)	adapter protein MecA	1.560	–
AEA93419.1 (*OG1RF_10732*)	FtsZ-interacting cell division protein YlmF	1.560	–
AEA94308.1 (*OG1RF_11621*)	NgoFVII restriction endonuclease superfamily protein	–	1.541
AEA94061.1 (*OG1RF_11374, buk*)	butyrate kinase	1.544	–
AEA94734.1 (*OG1RF_12047, spxA*)	regulatory protein Spx	–	1.524
AEA94693.1 (*OG1RF_12006, groEL*)	chaperonin GroEL	–	1.490
AEA93199.1 (*OG1RF_10512*)	MutT/NUDIX family protein	1.524	–
AEA94670.1 (*OG1RF_11983, murA2*)	UDP-N-acetylglucosamine 1-carboxyvinyltransferase	1.520	1.849
AEA93452.1 (*OG1RF_10765, drrC*)	daunorubicin resistance protein	1.507	1.310
AEA94047.1 (*OG1RF_11360, xerC*)	tyrosine recombinase XerC	1.500	–
AEA94288.1 (*OG1RF_11601*)	GNAT family acetyltransferase	1.497	–
AEA94227.1 (*OG1RF_11540*)	PP-loop family protein	1.496	1.546
AEA93700.1 (*OG1RF_11013*)	GntR family transcriptional regulator	1.478	–
AEA93154.1 (*OG1RF_10467*)	putative thioredoxin	–	1.475
AEA94591.1 (*OG1RF_11904, rimI*)	ribosomal-protein-alanine acetyltransferase	–	1.473
AEA93907.1 (*OG1RF_11220, fbp*)	fructose-1,6-bisphosphatase	1.455	1.600
AEA93067.1 (*OG1RF_10380*)	Nitroreductase	–	1.453
AEA94513.1 (*OG1RF_11826*)	NifU family SUF system FeS assembly protein	1.422	–
AEA93553.1 (*OG1RF_10866*)	hypothetical protein/Thioredoxin_like	–	1.418
AEA95234.1 (*OG1RF_12547, mycA*)	myosin-cross-reactive antigen	1.414	–
AEA94616.1 (*OG1RF_11929*)	hypothetical protein	–	1.410
AEA94405.1 (*OG1RF_11718*)	hypothetical protein	1.409	–
AEA93483.1 (*OG1RF_10796*)	hypothetical protein	1.405	–
AEA93230.1 (*OG1RF_10543*)	hypothetical protein	1.395	–
AEA95035.1 (*OG1RF_12348*)	GNAT family acetyltransferase	1.394	–
AEA95136.1 (*OG1RF_12449*)	M protein trans-acting positive regulator	1.392	–
AEA94760.1(*OG1RF_12073, recX*)	recombination regulator RecX	1.389	–
AEA94771.1(*OG1RF_12084*)	ABC superfamily ATP binding cassette transporter, ABC protein	1.385	–
AEA92793.1 (*OG1RF_10106*)	Oxidoreductase	1.384	–
AEA93715.1 (*OG1RF_11028*)	haloacid dehalogenase family hydrolase	–	1.368
AEA95260.1 (*OG1RF_12573*)	GntR family transcriptional regulator	1.362	–
AEA93441.1 (*OG1RF_10754*)	GNAT family acetyltransferase	1.361	–
AEA94573.1(*OG1RF_11886*)	hypothetical protein	1.357	–
AEA93214.1 (*OG1RF_10527*)	ABC superfamily ATP binding cassette transporter, ABC protein	1.353	–
AEA92756.1 (*OG1RF_10069*)	hypothetical protein	1.350	–
AEA93410.1(*OG1RF_10723*)	cell division protein	1.327	–
AEA94176.1 (*OG1RF_11489, purD*)	phosphoribosylamine-glycine ligase	1.316	–
AEA93340.1 (*OG1RF_10653*)	response regulator	1.316	–
AEA93256.1 (*OG1RF_10569*)	L-seryl-tRNA (Sec) selenium transferase	1.314	–
AEA94441.1 (*OG1RF_11754, gloA6*)	lactoylglutathione lyase	1.300	–
AEA94128.1 (*OG1RF_11441, aroD*)	3-dehydroquinate dehydratase	–	1.297
AEA94544.1 (*OG1RF_11857*)	GntR family transcriptional regulator	1.296	–
AEA94273.1 (*OG1RF_11586*)	transcriptional regulator	1.294	–
AEA92934.1 (*OG1RF_10247, gloA*)	lactoylglutathione lyase	1.290	–
AEA95077.1 (*OG1RF_12390, rpoZ*)	DNA-directed RNA polymerase subunit omega	–	1.283
AEA92957.1 (*OG1RF_10270*)	hypothetical protein	1.276	–
AEA94962.1 (*OG1RF_12275, agxt*)	Aminotransferase	1.276	–
AEA94536.1 (*OG1RF_11849, zurR*)	Fur family transcriptional regulator ZurR	1.271	–
AEA93513.1 (*OG1RF_10826*)	hypothetical protein	1.268	–
AEA93713.1 (*OG1RF_11026*)	protein of hypothetical function DUF1212	1.263	1.278
AEA94801.1 (*OG1RF_12114*)	ABC superfamily ATP binding cassette transporter, ABC protein	1.261	–
AEA93430.1 (*OG1RF_10743*)	sigma-54 interaction domain protein	–	1.261
AEA94727.1 (*OG1RF_12040, ppnK*)	NAD(+) kinase	1.261	–
AEA94097.1 (*OG1RF_11410*)	MerR family transcriptional regulator	1.258	–
AEA93950.1 (*OG1RF_11263*)	hypothetical protein	1.257	–
AEA94980.1 (*OG1RF_12293*)	M protein trans-acting positive regulator	–	1.257
AEA92806.1 (*OG1RF_10119, add*)	adenosine deaminase	1.255	–
AEA93450.1 (*OG1RF_10763*)	endonuclease/exonuclease/phosphatase	–	1.291
AEA94787.1 (*OG1RF_12100*)	Exonuclease	–	1.453
AEA94745.1 (*OG1RF_12058, sbcC*)	exonuclease SbcC	1.253	1.218
AEA95129.1 (*OG1RF_12442*)	FMN reductase	–	1.255
AEA93282.1 (*OG1RF_10595, opuAA*)	glycine betaine/L-proline ABC superfamily ATP binding cassette transporter, ABC protein	–	1.253
AEA93239.1 (*OG1RF_10552, rplY*)	50S ribosomal protein L25	1.246	–
AEA93267.1 (*OG1RF_10580*)	PemK family transcriptional regulator	–	1.252
AEA95167.1 (*OG1RF_12480*)	DEAH-box family ATP-dependent helicase	1.245	–
AEA94265.1 (*OG1RF_11578*)	alpha-hemolysin-like protein	–	1.249
AEA92804.1 (*OG1RF_10117*)	lipase/acylhydrolase	–	1.247
AEA93429.1 (*OG1RF_10742*)	DEAD/DEAH box family ATP-dependent RNA helicase	1.243	–
AEA92904.1 (*OG1RF_10217*)	phosphoglycerate mutase	1.241	–
AEA93066.1 (*OG1RF_10379*)	phage integrase family site-specific recombinase	–	1.238
AEA94914.1 (*OG1RF_12227*)	HAD superfamily hydrolase	1.237	–
AEA95081.1 (*OG1RF_12394*)	YicC like protein	1.236	–
AEA94216.1 (*OG1RF_11529, fsrA*)	FsrA response regulator	–	1.235
AEA93148.1 (*OG1RF_10461, gatC*)	glutamyl-tRNA (Gln) amidotransferase subunit C	–	1.234
AEA94809.1 (*OG1RF_12122, yaaT*)	stage 0 sporulation protein YaaT	–	1.234
AEA94698.1 (*OG1RF_12011*)	ABC superfamily ATP binding cassette transporter, ABC protein	1.231	–
AEA94715.1 (*OG1RF_12028, coaC*)	phosphopantothenoylcysteine decarboxylase	1.230	–
AEA93059.1 (*OG1RF_10372, pgpA*)	phosphatidylglycerophosphatase A	1.229	–
AEA94099.1 (*OG1RF_11412*)	transcriptional regulator	1.223	–
AEA93605.1 (*OG1RF_10918*)	MutT/NUDIX family protein	–	1.222
AEA93586.1 (*OG1RF_10899, murI*)	glutamate racemase	1.217	–
AEA92699.1 (*OG1RF_10012, dnaB*)	replicative DNA helicase DnaB	1.216	–
AEA94183.1 (*OG1RF_11496, purS*)	phosphoribosylformylglycinamidine synthase subunit PurS	1.215	–
AEA94204.1 (*OG1RF_11517, agaS*)	sugar isomerase protein AgaS	1.215	–
AEA94381.1 (*OG1RF_11694*)	ABC superfamily ATP binding cassette transporter, ABC protein	1.213	–
AEA92898.1 (*OG1RF_10211, dus*)	tRNA-dihydrouridine synthase	–	1.213
AEA93779.1 (*OG1RF_11092*)	ABC superfamily ATP binding cassette transporter, membrane protein	1.212	–
AEA94888.1 (*OG1RF_12201*)	D-isomer specific 2-hydroxyacid dehydrogenase	1.212	–
AEA93602.1 (*OG1RF_10915*)	2,5-diketo-D-gluconate reductase	1.212	–
AEA94423.1 (*OG1RF_11736*)	group 2 glycosyl transferase	1.211	–
AEA94516.1 (*OG1RF_11829, sufC*)	ABC superfamily ATP binding cassette transporter, ABC protein	–	1.208
AEA93609.1 (*OG1RF_10922*)	hypothetical protein	–	1.204
AEA93584.1 (*OG1RF_10897*)	glutamine ABC superfamily ATP binding cassette transporter, binding protein	1.200	–
Abundance-decreased			
AEA92854.1 (*OG1RF_10167, rplR*)	50S ribosomal protein L18	0.828	–
AEA93526.1 (*OG1RF_10839*)	universal stress protein	–	0.826
AEA92839.1 (*OG1RF_10152, rplD*)	ribosomal protein L4/L1 family protein	0.825	–
AEA95174.1 (*OG1RF_12487, rplM*)	50S ribosomal protein L13	0.816	–
AEA95058.1 (*OG1RF_12371, acpP2*)	acyl carrier protein	0.797	–
AEA93354.1 (*OG1RF_10667*)	hypothetical protein	0.794	–
AEA93187.1 (*OG1RF_10500*)	ATP-binding protein	–	0.792
AEA93783.1 (*OG1RF_11096*)	hypothetical protein	0.787	–
AEA93274.1 (*OG1RF_10587*)	hypothetical protein	–	0.787
AEA94998.1 (*OG1RF_12311, traC2*)	peptide ABC superfamily ATP binding cassette transporter, binding protein	0.776	–
AEA93330.1 (*OG1RF_10643, rplT*)	50S ribosomal protein L20	0.773	–
AEA94110.1 (*OG1RF_11423, pyrE*)	orotate phosphoribosyltransferase	–	0.763
AEA94119.1 (*OG1RF_11432, upp*)	uracil phosphoribosyltransferase	–	0.747
AEA94115.1 (*OG1RF_11428, carA*)	carbamoyl-phosphate synthase, small subunit	–	0.719
AEA92893.1 (*OG1RF_10206*)	S1 RNA-binding domain protein	0.705	–
AEA92696.1 (*OG1RF_10009, rpsR*)	30S ribosomal protein S18	0.671	–
AEA95155.1 (*OG1RF_12468, rpsN2*)	30S ribosomal protein S14	–	0.671
AEA94111.1 (*OG1RF_11424, pyrF*)	orotidine-5′-phosphate decarboxylase	0.670	–
AEA94116.1 (*OG1RF_11429, pyrC*)	Dihydroorotase	0.650	–
AEA94767.1 (*OG1RF_12080, rplL2*)	ribosomal protein L7/L12	0.649	–
AEA93420.1 (*OG1RF_10733, ylmG*)	YlmG protein	0.579	–
AEA94625.1 (*OG1RF_11938*)	fumarate reductase	0.565	–
AEA93191.1 (*OG1RF_10504*)	thioredoxin superfamily protein	0.493	0.289
AEA93192.1 (*OG1RF_10505, clpP*)	ATP-dependent Clp protease proteolytic subunit	0.385	0.337

The data are given as the means of the results from two independent experiment. *WT* wild-type/parent strain; −, 0.83 ≤ △*clpP*/WT ratio ≤ 1.2

### Gene ontology (GO) and Kyoto encyclopedia of genes and genomes (KEGG) analysis of DAPs

**Fig. 6:**
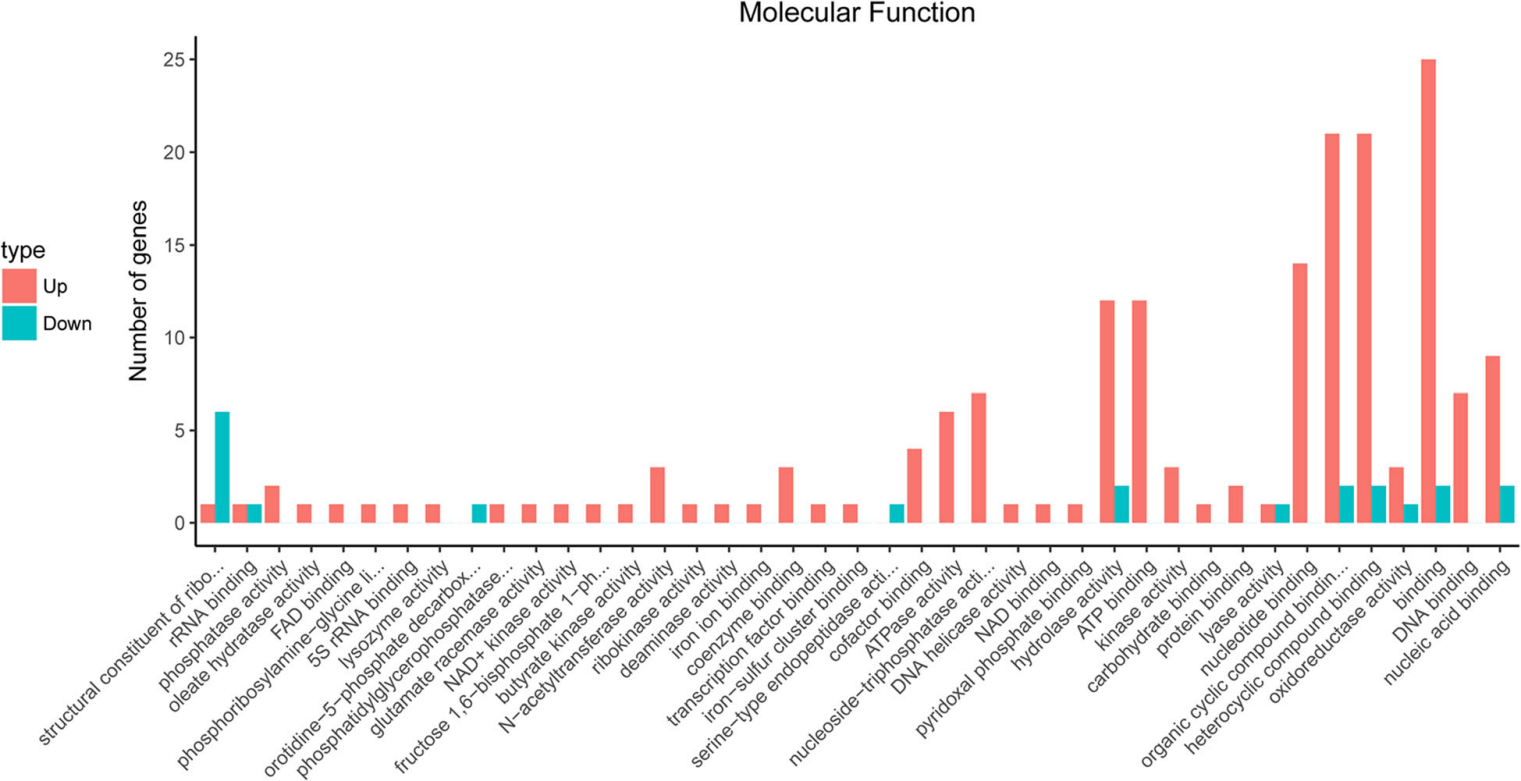
Gene Ontology (GO) analysis of differential abundance proteins (DAPs). The molecular functions of DAPs were classified by GO analysis

**Fig. 7:**
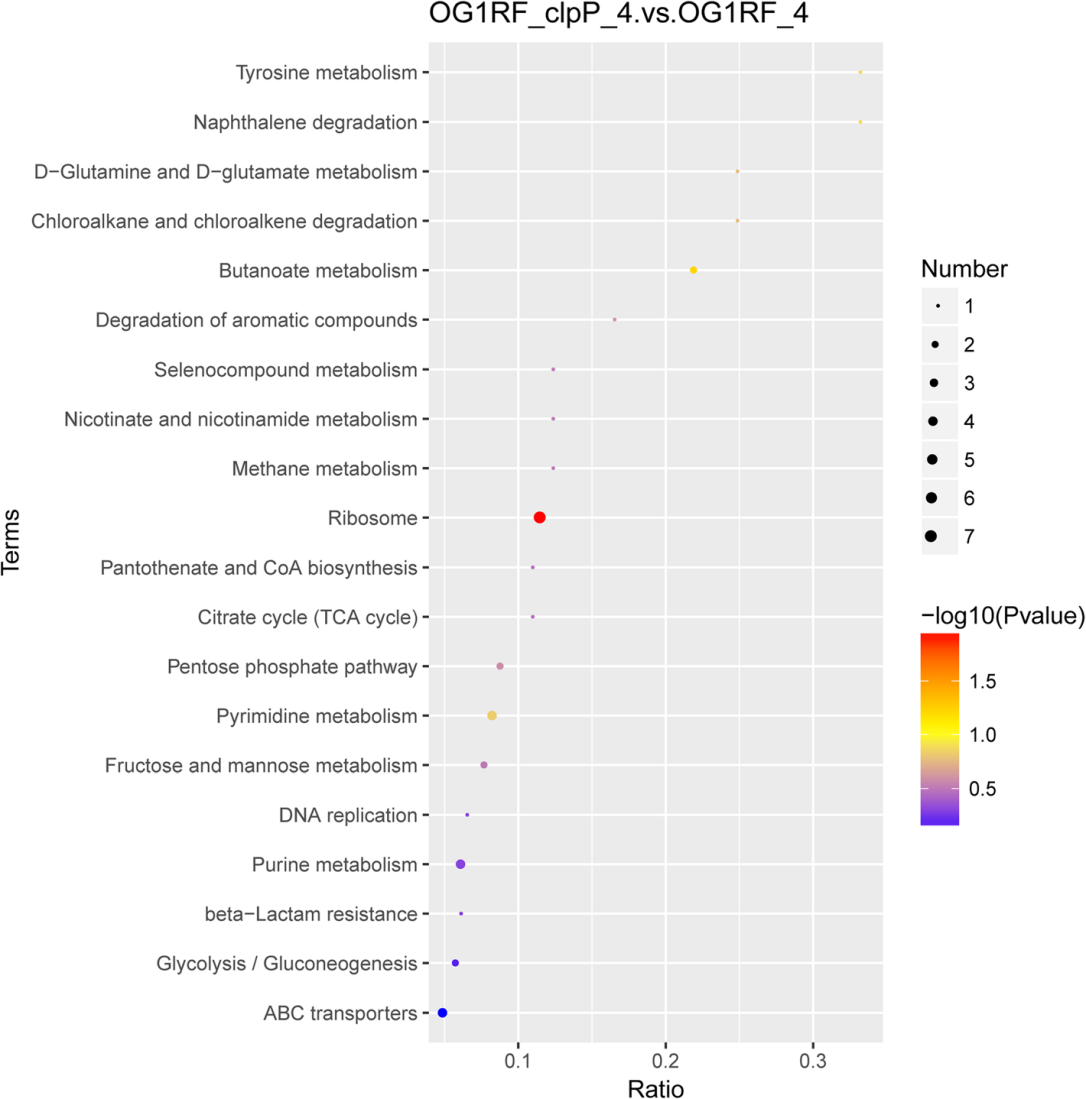
KEGG (Kyoto Encyclopedia of Genes and Genomes) analysis of differential abundance proteins (DAPs) (logarithmic phase). The protein families and pathways were analyzed using the KEGG database

DAPs between the △*clpP* mutant and parent strain were analyzed by GO and KEGG analyses. As shown in Fig. 6, GO analysis revealed that increased DAPs in the △*clpP* mutant strain (logarithmic phase) were mainly concentrated in the following molecular functions: N-acetyltransferase activity, coenzyme binding, cofactor binding, ATPase activity, nucleoside-triphosphatase activity, hydrolase activity, ATP binding, kinase activity, nucleotide binding, organic cyclic compound binding, heterocyclic compound binding, DNA binding, and nucleic acid binding. Decreased DAPs were mainly included in the following molecular functions: structural constituent of ribosome, rRNA binding, orotidine-5′-phosphate decarboxylase activity, hydrolase activity, organic cyclic compound binding, heterocyclic compound binding, and nucleic acid binding. KEGG analysis demonstrated that the functions of most DAPs in the △*clpP* mutant (logarithmic phase) belonged to the ribosome, fructose and mannose metabolism, pyrimidine metabolism, purine metabolism, pentose phosphate pathway, glycolysis/gluconeogenesis, and ABC transporters (Fig. 7). The functions of DAPs in the stationary phase of △*clpP* mutant strain were similar to those in the logarithmic phase (Additional file 3: Figure S3).

### DAPs associated with the stress response, virulence, or biofilm formation of *E. faecalis*

Based on the literature, we selected DAPs that may be associated with stress response, virulence, or biofilm formation of *E. faecalis* for a thorough analysis. The abundance of DAPs associated with the stress response or virulence of *E. faecalis* increased in the △*clpP* mutant strain, including the FsrA response regulator and gelatinase GelE; ATPase/chaperone ClpC; chaperonin GroEL, acetyl esterase/lipase; and transcriptional regulator proteins, HrcA, CtsR, and Spx (Table 2**).** However, the abundances of ribosomal proteins L4/L1, L7/L12, L13, L18, L20, S14, and S18 decreased in the △*clpP* mutant strain. The abundance of the biofilm formation of *E. faecalis-*associated DAPs and adapter protein MecA increased in the △*clpP* mutant strain, while the abundances were lower for orotate phosphoribosyltransferase, orotidine-5′-phosphate decarboxylase, and dihydroorotase (Table 2**)**. The RNA levels of all the above DAPs were verified by RT-qPCR and were consistent with protein abundance changes in the △*clpP* mutant strain.

**Table 2:** Differential abundance proteins associated with the stress response or virulence, biofilm formation of *E. faecalis*

Protein ID (locus_tag, gene name)	Description or predicted function	Protein abundance ratio of △*clpP*/WT^a^		RNA ratio of △*clpP*/WT (RT-qPCR)^b^	
		4 h	12 h	4 h	12 h
Stress response or virulence					
AEA94216.1 (*OG1RF_11529, fsrA*)	FsrA response regulator	–	1.235	0.965	1.162
AEA94213.1 (*OG1RF_11526*, *gelE*)	gelatinase GelE	–	2.577	1.267	2.587
AEA95218.1 (*OG1RF_12531*, *ctsR*)	transcriptional regulator CtsR	2.165	1.894	2.024	2.365
AEA95217.1 (*OG1RF_12530, clpC*)	ATPase/chaperone ClpC	1.906	1.802	2.345	1.687
AEA94933.1(*OG1RF_12246*)	Acetyl esterase/lipase	–	1.873	1.368	1.928
AEA94734.1 (*OG1RF_12047, spxA*)	regulatory protein Spx	–	1.524	1.258	1.834
AEA93763.1(*OG1RF_11076, hrcA*)	heat-inducible transcription repressor HrcA	–	1.692	1.267	2.364
AEA94693.1 (*OG1RF_12006, groEL*)	chaperonin GroEL	–	1.490	0.831	1.859
AEA92854.1 (*OG1RF_10167, rplR*)	50S ribosomal protein L18	0.828	–	0.768	1.227
AEA92839.1 (*OG1RF_10152, rplD*)	ribosomal protein L4/L1 family protein	0.825	–	0.657	0.948
AEA95174.1 (*OG1RF_12487, rplM*)	50S ribosomal protein L13	0.816	–	0.584	1.168
AEA93330.1 (*OG1RF_10643, rplT*)	50S ribosomal protein L20	0.773	–	0.408	0.862
AEA92696.1 (*OG1RF_10009, rpsR*)	30S ribosomal protein S18	0.671	–	0.352	1.358
AEA95155.1 (*OG1RF_12468, rpsN2*)	30S ribosomal protein S14	–	0.671	0.862	0.518
AEA94767.1 (*OG1RF_12080, rplL2*)	ribosomal protein L7/L12	0.649	–	0.338	1.168
Biofilm formation					
AEA94733.1 (*OG1RF_12046, mecA*)	adapter protein MecA	1.560	–	2.984	1.537
AEA94110.1 (*OG1RF_11423, pyrE*)	orotate phosphoribosyltransferase	–	0.763	0.867	0.658
AEA94111.1 (*OG1RF_11424, pyrF*)	orotidine-5′-phosphate decarboxylase	0.670	–	0.318	0.834
AEA94116.1 (*OG1RF_11429, pyrC*)	dihydroorotase	0.650	–	0.495	1.162

^a^The data are given as the means of the results from two independent experiment. ^b^The data are given as the means of the results from three independent experiment. *WT* wild-type/parent strain; −, 0.83 ≤ △*clpP*/WT ratio ≤ 1.2

## Discussion

ClpP is a protease of the Hsp100/Clp family that is very important for bacterial growth and plays an irreplaceable role in cellular protein quality control systems by refolding or degrading damaged proteins in stressed cells [14]. To date, ClpP has been implicated in many essential bacterial activities such as stress responses to abnormal temperature, hyperosmotic pressure, low pH, oxidative stress, virulence, and biofilm formation. However, the global abundances of proteins affected by ClpP in bacteria are still little known. Feng et al. found that the abundance of transcriptional regulators CtsR and Spx, the ClpC adaptor proteins McsB and MecA, and the cell division protein FtsZ were clearly affected by ClpP in *S. aureus* strains NCTC8325–4, COL, SA564, and Newman using a two-dimensional difference gel electrophoresis (2-D DIGE) technique [29, 30]. However, the abundances of only 80 proteins changed in their studies, a result that may be due to the low sensitivity of 2-D DIGE. In the present study, we found 135 DAPs in the △*clpP* mutant strain. These included the transcriptional regulators CtsR and Spx, the ClpC adaptor proteins MecA and FtsZ-interacting cell division protein YlmF, as previously described in *S. aureus* strains. Interestingly we also found other new proteins, such as acetyl esterase/lipase, ribosomal protein, orotidine-5′-phosphate decarboxylase, and others.

ClpP has been shown to participate in stress tolerance by refolding or degrading damaged proteins during bacteria growth, and several studies have indicated that the Δ*clpP* mutant strain showed a growth defect over a broad range of temperatures including high (40, 42, 45 °C) or low (20, 30 °C) temperatures, and even under 37 °C [19, 23, 31, 32]. However, this study showed altered growth of *E. faecalis* OG1RF Δ*clpP* mutant strain at 45 °C and 20 °C but not 37 °C. Previous studies also demonstrated the Δ*clpP* strain is more vulnerable to oxidative stress, osmotic stress, acid, or sodium dodecyl sulfate (SDS) [19, 33–35]. We found the growth of OG1RF Δ*clpP* was impaired under osmotic or oxidative stress conditions. The ribosomal protein L9 plays a significant role in the *Escherichia coli* response to starvation stress [36]. The present study found that in *E. faecalis* OG1RF, the abundance of many ribosomal proteins decreased, including both 50S and 30S ribosomal proteins. Thus, ClpP may participate in the stress response of *E. faecalis* by affecting the abundance of ribosomal proteins*.*

Previous studies have found that ClpP can significantly affect bacteria biofilm formation, but its effects in different genera vary [15, 16, 18, 19, 21]. This study provides the first evidence that biofilm formation decreased when the *clpP* of OG1RF strain was deleted. The adapter protein MecA can decrease the RNA level of *eps*, which encodes synthesis of the biofilm matrix exopolysaccharide, thus inhibiting biofilm formation by *Bacillus subtilis* [37]. The present study showed MecA abundance increased in the Δ*clpP* mutant strain, and this contribute to the decreased biofilm formation of the *clpP* deleted strain. Another reason for decreased biofilm formation of the Δ*clpP* mutant strain may be the reduced abundances of orotate phosphoribosyltransferase (*pyrE*) and orotidine-5′-phosphate decarboxylase (*pyrF*), proteins that promote the biofilm formation of *Streptococcus sanguinis* and *E. faecalis*, respectively [38, 39].

ClpP participates in bacterial virulence, and the virulence of *S. pneumoniae*, *S. aureus* and *L. pneumophila* was attenuated in *clpP* mutation strains [22–24]. Liu et al. *recently* reported that the *clpP* mutant strain showed increased biofilm formation and reduced virulence in *S. aureus* [21]. However, we found that the Δ*clpP* mutant strain decreased biofilm formation and increased virulence in a *G. mellonella* model. A previous study proposed that the CtsR regulator controlled the expression of *clpC*, *clpE,* and *clpP* and was required for the virulence of *E. faecalis* V583, but the role of *clpP* in the virulence of *E. faecalis* was still unclear [40]. The FsrABDC signal transduction system and GelE are major virulence factors in *E. faecalis* [41, 42]. Thus, it may be that the increased abundances of FsrA and GelE leads enhance virulence of the Δ*clpP* mutant strain. The abundance of acetyl esterase/lipase, another *E. faecalis* virulence factor, was also increased in the Δ*clpP* mutant strain and may contribute to the enhanced virulence of the Δ*clpP* mutant strain [43].

This study also found that the tolerance to linezolid or minocycline of the Δ*clpP* mutant strain decreased. Linezolid is an inhibitor of bacterial protein synthesis that acts on the 50S ribosome subunit of gram-positive bacteria, and minocycline is a synthetic tetracycline derivative that acts on the 30S ribosome subunit of gram-positive or -negative bacteria [44, 45]. The abundances of 50S ribosomal proteins L13, L18, and L20 and 30S ribosomal proteins S14 and S18 were decreased in the △*clpP* mutant strain, thus might lead to the decrease of the tolerance of the △*clpP* mutant strain to linezolid or minocycline.

In *B. subtilis*, Spx plays a significant role in protecting against oxidative stresses [46]. Recently Rojas-Tapias and Helmann found that Spx is a regulator of the ctsR operon, and the ctsR operon regulates the expression of *clpC* and *clpP* [47]. The present study showed that when *clpP* was deleted in *E. faecalis* OG1RF, the abundance of ClpC, CtsR, and Spx all increased, which was similar to observations in *S. aureus* [30]. In *S. aureus,* the RNA levels of the *clpC* operon (*ctsR*-*mcsA*-*mcsB*-*clpC*), *groE*, and *dnaK* were induced in response to accumulation of misfolded proteins, which supported the hypothesis that ClpP proteases degrade misfolded proteins [30]. Our study found that the abundances of ClpC, GroEL, and DnaB (but not DnaK) increased in the △*clpP* mutant strain, possibly due to the accumulation of misfolded proteins.

It is easy to understand how ClpP, as a protease, can significantly affect the abundance of proteins, but not RNA levels. In the present study, the abundances of many transcription regulation-related proteins changed in the △*clpP* mutant strain, such as regulatory protein Spx (spxA), heat-inducible transcription repressor HrcA, transcriptional regulator CtsR, as reported previously [29, 30]. Transcriptional regulators usually control the transcription and RNA levels of their functional genes. So, ClpP may affect the abundance of transcriptional regulators alter the RNA levels of the genes. The RNA levels of many genes changed in the Δ*clpP* mutant strain in this study, and similar results were reported in other studies [23, 30]. Since ClpP is a protease involved in protein degradation, its absence should lead to protein accumulation, and this is consistent with our result that the abundance of most DAPs increased in the △*clpP* mutant strain. However, the abundances of some proteins and their corresponding RNA levels decreased in the △*clpP* mutant strain, and similar results were also found in another study [30]. As mentioned above, the reason may be that ClpP reduced the transcription and expression of those genes by regulating the abundance of transcriptional regulators.

## Conclusion

The present study indicates that ClpP may affect the abundance of ribosomal proteins L4/L1, L7/L12, L13, L18, L20, S14, and S18 that participate in the stress response and linezolid or minocycline tolerance of *E. faecalis*. ClpP participates in *E. faecalis* biofilm formation by affecting the abundances of adapter protein MecA, orotate phosphoribosyltransferase (*pyrE*), and orotidine-5′-phosphate decarboxylase (*pyrF*). Our results also suggest that ClpP may modulate the abundances of FsrA, GelE, and acetyl esterase/lipase to participate in *E. faecalis* virulence.

## Methods

### Bacterial strains, plasmids, growth conditions, and chemicals

All of the bacterial strains and plasmids used in this study are shown in Table 3. *E. faecalis* ATCC 47077 (OG1RF; GenBank accession number CP002621.1) and ATCC 29212 were purchased from the American Type Culture Collection (ATCC; Manassas, VA, USA). *E. faecalis* strains were cultured in tryptic soya broth (TSB; Oxoid, Basingstoke, UK) as previously described [28]. TSBG (TSB medium added 0.25% glucose) for biofilm formation detection. Electroporation was used for plasmid transformation, and B2 medium was used for bacteria recovery [28]. The antibiotics used in this study were purchased from Sigma Chemical Co. (St Louis, MO, USA) and used at concentrations of 20 mg/L for chloramphenicol and 750 or 25 mg/L for erythromycin.

**Table 3:** Bacterial strains and plasmids used in the present study

Bacterial strain or plasmid	Description	Source
Bacterial strains		
*E.faecalis* OG1RF	Rifampin- and fusidic acid-resistant derivative of human oral cavity isolate	ATCC
△*clpP* mutant	*clpP* deletion mutant obtained using OG1RF as the parent strain	This study
△*clpP/*pIB166::*clpP*	*clpP* deletion mutant complemented with plasmid pIB166 harboring the *clpP* gene	This study
△*clpP/* pIB 166	*clpP* deletion mutant complemented with plasmid pIB166	This study
*E.faecalis* ATCC29212	Standard strain for the MIC detection	ATCC
Plasmids		
pJRS233	Temperature-sensitive *E. coli* (Erm^750^)-*Enterococcus* (Erm^25^) shuttle vector	Michael G. Caparon, Washington University
pJRS233-△*clpP*	Temperature-sensitive plasmid for generation of in-frame deletion of *clpP*	This study
pIB166	*E. coli* (Cm^20^)-*Streptococcus* (Cm^20^) shuttle vector	Jingren Zhang, Tsinghua University
pIB166::*clpP*	Used for complementation of *clpP* deletion	This study

### Construction of △*clpP* mutants and complemented strains

The *clpP* deletion mutant of the OG1RF strain was constructed by in-frame deletion using the temperature-sensitive plasmid pJRS233 as previously described [48]. Briefly, the upstream and downstream fragments of *OG1RF_10505* (gene: *clpP*; product: ATP-dependent Clp protease proteolytic subunit), which is highly homologous (86.8%) to *SA0723* (product as the ClpP protease) of *S. aureus* N315 strain [23], were amplified from OG1RF by PCR and separately cloned into the pJRS233 vector to generate pJRS233-Δ*clpP*. The recombinant plasmid pJRS233-Δ*clpP* was successively transferred and electroporated into wildtype OG1RF strain, then the pJRS233-Δ*clpP* clones were selected by variable temperature screening as previously described [28]. The complemented Δ*clpP* mutant strain was constructed using the *E. coli* -*Streptococcus* shuttle vector pIB166. The *clpP* gene was amplified by PCR and cloned into the pIB166 vector to produce pIB166:: *clpP*. The recombinant plasmid pIB166:: *clpP* was transformed by electroporation into the Δ*clpP* mutant strain, forming the complemented Δ*clpP*/pIB166:: *clpP* strain. The Δ*clpP* strain containing the empty vector pIB166 was designated the Δ*clpP*/pIB166 mutant. The Δ*clpP* mutant and complemented Δ*clpP* mutant strain were identified by PCR, RT-qPCR, and direct sequencing. The primers used in this assay are listed in Table 4.

**Table 4:** Primers used for △*clpP* mutant construction and complemented strains

Primers^a^	Sequences (5′ → 3′)	Location^b^	PCR product size (bp)	Note^c^
Construction of Δ*clpP* mutant				
*clpP* us-F	GCTCTAGATTTGGGGTGTTTGTTTAGCAG	530,796–530,816	1059	XbaI
*clpP* us-R	CGGGATCCGGAAGTAAATCCTCCTATATAAAG	529,774–529,794		BamHI
*clpP* ds-F	CGGGATCCCAGCGACACTCGCTGTTTCCA	529,139–529,159	1044	BamHI
*clpP* ds-R	AACTGCAGAGGCGTAGATGAACCAGTGGT	528,132–528,152		PstI
Construction of Δ*clpP* complemented strain				
HB*clpP*-F	CGGGATCCAGGCATTCAAAGTGCTTTGTG	529,816–529,836	714	BamHI
HB*clpP*-R	CCCTCGAGTGGAAACAGCGAGTGTCGCTG	529,139–529,159		XhoI
RT-qPCR				
*recA*-F	CGACTAATGTCTCAAGCACTAC	2,574,587–2,574,608	106	
*recA*-R	CGAACATCACGCCAACTT	2,574,503–2,574,520		
*clpP*-F	TTAATTCCAACAGTTATTGAA	529,746–529,766	198	
*clpP*-R	ACCAGGAGAGTTAATGTA	529,569–529,586		

^*a*^Primers were designed according to the genomic sequence of *E. faecalis* OG1RF (GenBank accession number CP002621.1). F, forward primer; R, reverse primer. ^*b*^Location of the primer in the genomic sequence of *E. faecalis* OG1RF (GenBank accession number CP002621.1). ^*c*^The underlined sequences represent restriction enzyme sites

### Growth analysis of the △*clpP* mutant strain

The OG1RF, Δ*clpP*, Δ*clpP*/pIB166:: *clpP*, and Δ*clpP*/pIB166 strains were cultured in TSB at 37 °C with shaking for 12 h and diluted in the same medium to an OD_600_ value of 1.5, then 50 μL aliquot of the diluted suspension was inoculated into 10 mL fresh TSB and incubated at either 37 °C, 45 °C or 20 °C with circular agitation (220 rpm). The diluted suspension was also inoculated into fresh TSB with 5% NaCl pH 5.5 or 2 mM H_2_O_2_ and incubated at 37 °C with circular agitation (220 rpm). OD_600_ values for the cultures were determined using an Eppendorf Biospectrometer (Eppendorf, Hamburg, Germany) at 1-h intervals. Three independent experiments were performed.

### The sensitivity of the △*clpP* mutant strain to SDS

Overnight cultures of *E. faecalis* strains were diluted 1:200 in fresh TSB medium and incubated at 37 °C for 4 h until an OD_600_ of 1.0 was reached. After 10-fold serial dilution, 5 μL of the aliquot was spotted onto a TSB agar plate containing 0.008% SDS and incubated at 37 °C for 24 h. Bacterial colonies on the plates were photographed and counted [28]. Three independent experiments were performed, and representative results are shown.

### Microtiter plate assay of biofilm formation

The biofilm-forming ability of *E. faecalis* isolates was detected as previously described with modifications [49]. Overnight cultures were diluted 1:200 in 200 μL of TSBG (TSB with 0.25% glucose) and inoculated into 96-well polystyrene microtiter plates. After 12, 24, or 48 h of static incubation at 37 °C, the supernatant was discarded, and plates were washed thrice with deionized water to remove unattached cells, stained with 1% CV for 20 min at room temperature, and rinsed with distilled water. Finally, the CV was solubilized in ethanol-acetone (80:20, vol/vol), and absorbance at OD_570_ was determined. Three independent experiments were performed.

### Quantification of eDNA

eDNA was quantified as described previously [50]. Overnight cultures of *E. faecalis* strains were diluted to OD_600_ = 0.001 in AB medium supplemented with 0.5% glucose, 0.05 mM propidium iodide (PI) and 10% TSB. The diluted cultures were transferred to polystyrene microtiter plates (200 μL/well) and incubated for 24 h at 37 °C. The cell density was measured at OD_600_ using a microtiter plate reader (Bio-Rad Laboratories, Hercules, CA, USA). The fluorescence of PI-bound eDNA was measured by a Varioskan™ LUX multimode microplate reader (Thermo Fisher, Waltham, MA, USA) with the excitation/emission wavelength at 535/610 nm. Relative amounts of eDNA per OD_600_ unit were determined. Three independent experiments were performed.

### Determination of MIC and antimicrobial tolerance of strains

The MICs of the antimicrobials against *E. faecalis* isolates were determined by the broth microdilution method according to Clinical and Laboratory Standards Institute (CLSI) guideline CLSI-M100-S26 with CLSI-recommended MIC breakpoints. *E. faecalis* ATCC29212 served as the quality control standard strain. The antimicrobial-tolerance of strains was detected as described previously with modifications [28]. Antimicrobials (at 50× MIC) were added to the stationary-phase cultures (16 h) of the *E. faecalis* strains, then the cultures were incubated at 37 °C for 120 h without shaking. Every 24 h, 1-mL aliquots were sampled and washed twice with ice-cold saline. Ten-fold dilutions were then plated on Muller-Hinton agar, and the numbers of CFUs were determined. Three independent experiments were performed.

### Virulence of *E. faecalis* in G. mellonella

Infection of *G. mellonella* larvae with *E. faecalis* strains was performed as described previously for other pathogens [51]. *G. mellonella* larvae in groups of 40 were infected in the left posterior proleg with 20 μL inocula of *E. faecalis* strains containing 5 × 10^6^ CFU/mL. Survival of *G. mellonella* larvae was recorded at 12 h intervals for 72 h p.i. Every trial included a group of 20 *G. mellonella* larvae injected with saline as a control. Experiments were performed in at least three independent tests, and representative results are shown.

### Protein extraction and detection by a mass spectrometer with TMT labeling

*E. faecalis* strain OG1RF and the Δ*clpP* mutant were inoculated into TSB and cultured at 37 °C for 4 h to logarithmic phase or for 12 h to stationary phase. The cells were harvested at 4 °C centrifugation, minced individually with liquid nitrogen, lysed in lysis buffer, and ultrasonicated for 5 min on ice. Protein concentration was determined again with Bradford protein assays. The supernatant from each sample, containing precisely 0.1 mg of protein, was digested with Trypsin Gold (Promega, Madison, WI, USA) at 1:50 enzyme-to-substrate ratio. After 16 h of digestion at 37 °C, peptides were desalted with a C18 cartridge to remove urea, and desalted peptides were dried by vacuum centrifugation. Desalted peptides were labeled with TMT6/10-plex reagents (TMT6/10plex™ Isobaric Label Reagent Set, Thermo Fisher) as previously described [52]. TMT-labeled peptide mix was fractionated using a C18 column (Waters BEH C18 4.6 × 250 mm, 5 μm; Waters Corporation, Milford, MA, USA) on a Rigol L3000 high-performance liquid chromatographer operating at 1 mL/min, and the column oven was set at 50 °C. Shotgun proteomics analyses were performed using an EASY-nLCTM 1200 ultra high-performance liquid chromatography system (Thermo Fisher) coupled with an Orbitrap Q Exactive HF-X mass spectrometer (Thermo Fisher) operated in the data-dependent acquisition mode. The Q Exactive HF-X mass spectrometer was operated in positive polarity mode with a spray voltage of 2.3 kV and capillary temperature of 320 °C. Two independent experiments were performed.

### Global protein abundance analysis

The resulting spectra from each fraction were searched separately against the NCBI *E. faecalis* strains OG1RF (CP002621.1) database (https://www.ncbi.nlm.nih.gov/nuccore/CP002621.1) using the search engine Proteome Discoverer 2.2 (PD 2.2, Thermo). The searched parameters were as follows: mass tolerance of 10 ppm for precursor ion scans and mass tolerance of 0.02 Da for production scans. Carbamidomethyl was specified in PD 2.2 as a fixed modification. Oxidation of methionine, acetylation of the N-terminus, and TMT of lysine were specified in PD 2.2 as variable modifications. A maximum of 2 miscleavage sites was allowed. For protein identification, a protein with at least one unique peptide was identified at a false discovery rate FDR < 1.0% on peptide and protein levels. Proteins containing similar peptides that could not be distinguished based on MS/MS analysis were grouped as separate protein groups. The protein quantitation results were statistically analyzed by Mann-Whitney tests, and the significance ratios defined as *P* < 0.05 and ratio > 1.2 or < 0.83 (FC) were used to screen DAPs. GO and InterPro (IPR) analyses were conducted using the interproscan-5 program against the non-redundant protein database (including Pfam, PRINTS, ProDom, SMART, ProSiteProfiles, and PANTHER). The databases of COG (Clusters of Orthologous Groups) and KEGG were used to analyze protein families and pathways. The enrichment pipeline was used to perform the enrichment analyses of GO, IPR, and KEGG.

### RNA isolation and RT-qPCR

RNA isolation of *E. faecalis* strains was performed as described previously with some modifications [28]. The *E. faecalis* strain OG1RF and the Δ*clpP* mutant were inoculated into TSB and cultured at 37 °C for 4 h to logarithmic phase or for 12 h to stationary phase, and the following operations were performed at 4 °C for centrifugation or on ice. Bacterial cultures were centrifuged at 12,000 rpm for 5 min, and then the pellets were washed twice with 0.9% saline; the culture was homogenized 5 times using 0.1-mm zirconia-silica beads in a mini-BeadBeater (Biospec, Bartlesville, OK, USA) at 5000 rpm for 60 s at 1-min intervals; the samples were centrifuged at 15,000 rpm, and the bacterial RNA in the supernatant was purified using an RNeasy minikit (Qiagen, Hilden, Germany) and quantified using an ND-1000 spectrophotometer (NanoDrop Technologies, Wilmington, DE, USA). RNA samples that had a 260/280 ratio between 2.0 and 2.2 were used for RT-qPCR.

Total RNA extracted from strains OG1RF and the Δ*clpP* mutant were reverse transcribed with the PrimeScript RT Reagent Kit (TaKaRa Biotechnology, Dalian, China), and RT-qPCR was performed with the SYBR Premix Ex Taq II Kit (TaKaRa Biotechnology) on the Mastercycler ep realplex system (Eppendorf), with an initial incubation at 95 °C for 2 min, followed by 40 cycles of 15 s at 95 °C, and 60 s at 60 °C. Each sample was analyzed in triplicate. For all samples, the internal control gene *recA* was used to normalize the abundance of *E. faecalis* strains OG1RF genes [53]. The threshold cycle (Ct) numbers were confirmed by the detection system software, and the data were analyzed based on the 2^−△△Ct^ method. The RT-qPCR primers are listed in Additional file 4: Table S2.

### Statistical analysis

Experimental data were analyzed with SPSS software (version 16.0; SPSS, Chicago, IL, USA) and compared using Student’s t tests, one-way analysis of variance, Mann-Whitney tests, or the log-rank tests. Differences with a *P* value < 0.05 were considered statistically significant.

## Supplementary information

**Additional file 1: Figure S1.** Relative RNA levels of *clpP* in *E. faecalis* strains. The RNA levels of *clpP* were determined by RT-qPCR, with the OG1RF parent strain as the reference strain (RNA level = 1). Three independent experiments were performed, and the data represent means ± SD.

**Additional file 2: Figure S2.** Coefficient of variation (CV) distributions for the two independent repetitions of the four group samples. The proteins were extracted from the *E. faecalis* OG1RF and its Δ*clpP* mutant strains and divided into four groups: strains cultured at 37 °C for 4 h to logarithmic phase were marked as OG1RF_4 or OG1RF_clpP_4; strains cultured at 37 °C for 12 h to stationary phase were marked as OG1RF_12 or OG1RF_clpP_12. The proteins extracted from each group included two independent biological repetitions, and the peptides were labeled with TMT6/10-plex reagents, then sequenced with the Orbitrap Q Exactive HF-X mass spectrometer.

**Additional file 3: Figure S3.** Kyoto Encyclopedia of Genes and Genomes (KEGG) analysis of differential abundance proteins (DAPs) (stationary phase). The protein family and pathway were analyzed using the KEGG database.

**Additional file 4: Table S1.** Antimicrobial susceptibility of *E.faecalis* determined by conventional broth macrodilution method. **Table S2.** Primers used for the RT-qPCR for the detection of RNA levels of differential abundance proteins.

## Data Availability

All data generated or analyzed during this study are included in this published article [and its supplementary information files]. The mass spectrometry proteomics data were deposited to the ProteomeXchange Consortium via the PRIDE partner repository with the dataset identifier PXD014211.

## Abbreviations

CV: Crystal violet
DAP: Differential abundance protein
eDNA: Extracellular DNA
MIC: Minimal inhibitory concentration
TMT: Tandem mass tags
VRE: Vancomycin-resistant enterococci
