# Supplementary figures and images for: ClpP participates in stress tolerance, biofilm formation, antimicrobial tolerance, and virulence of Enterococcus faecalis
Source: BMC Microbiol. 2020 Feb 7;20:30. doi: 10.1186/s12866-020-1719-9 (PMC7006429; doi:10.1186/s12866-020-1719-9)

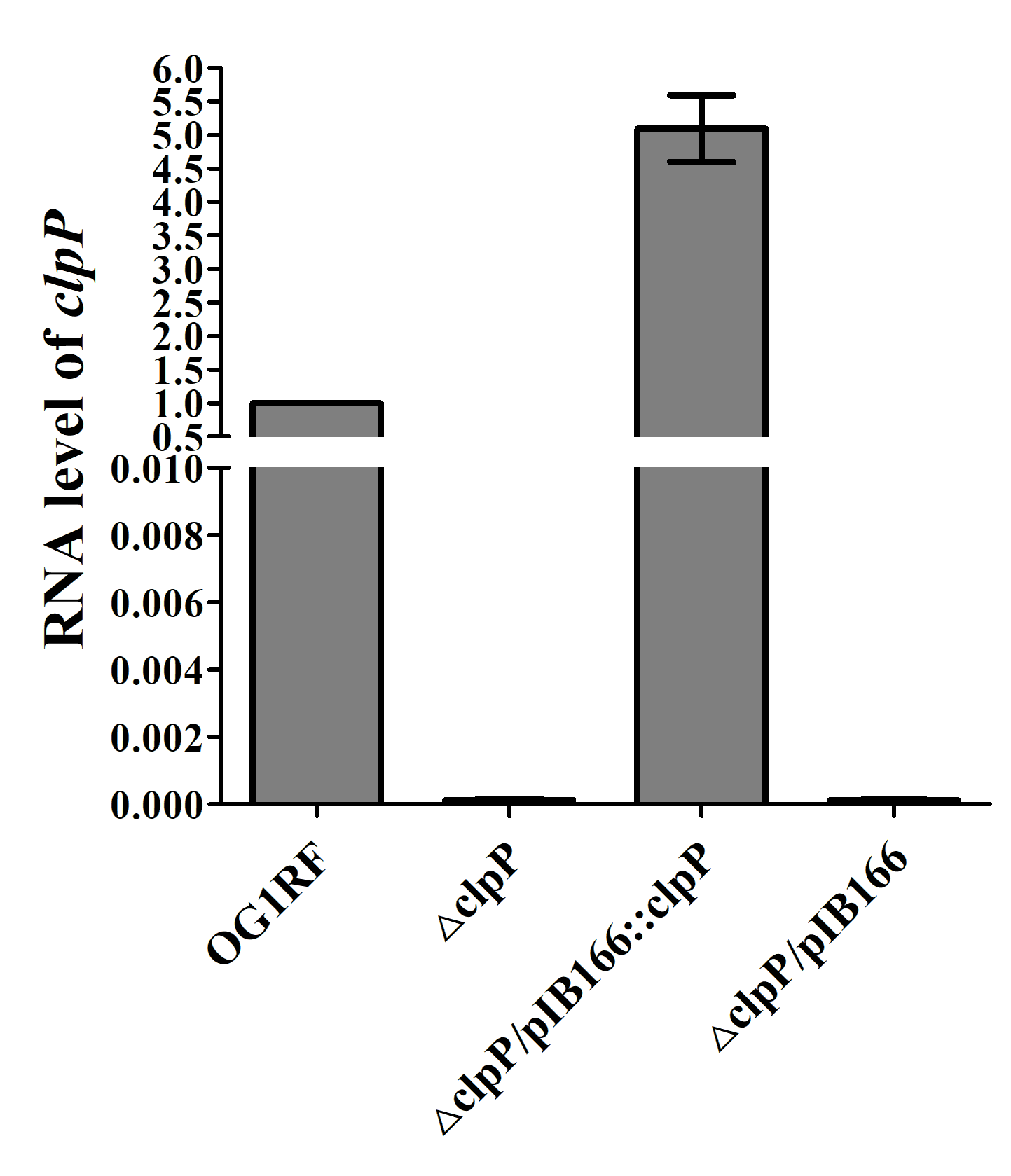

Supplement: Supplementary file 1 — Additional file 1: Figure S1. Relative RNA levels of clpP in E. faecalis strains. The RNA levels of clpP were determined by RT-qPCR, with the OG1RF parent strain as the reference strain (RNA level = 1). Three independent experiments were performed, and the data represent means ± SD. [file 12866_2020_1719_MOESM1_ESM.tif]

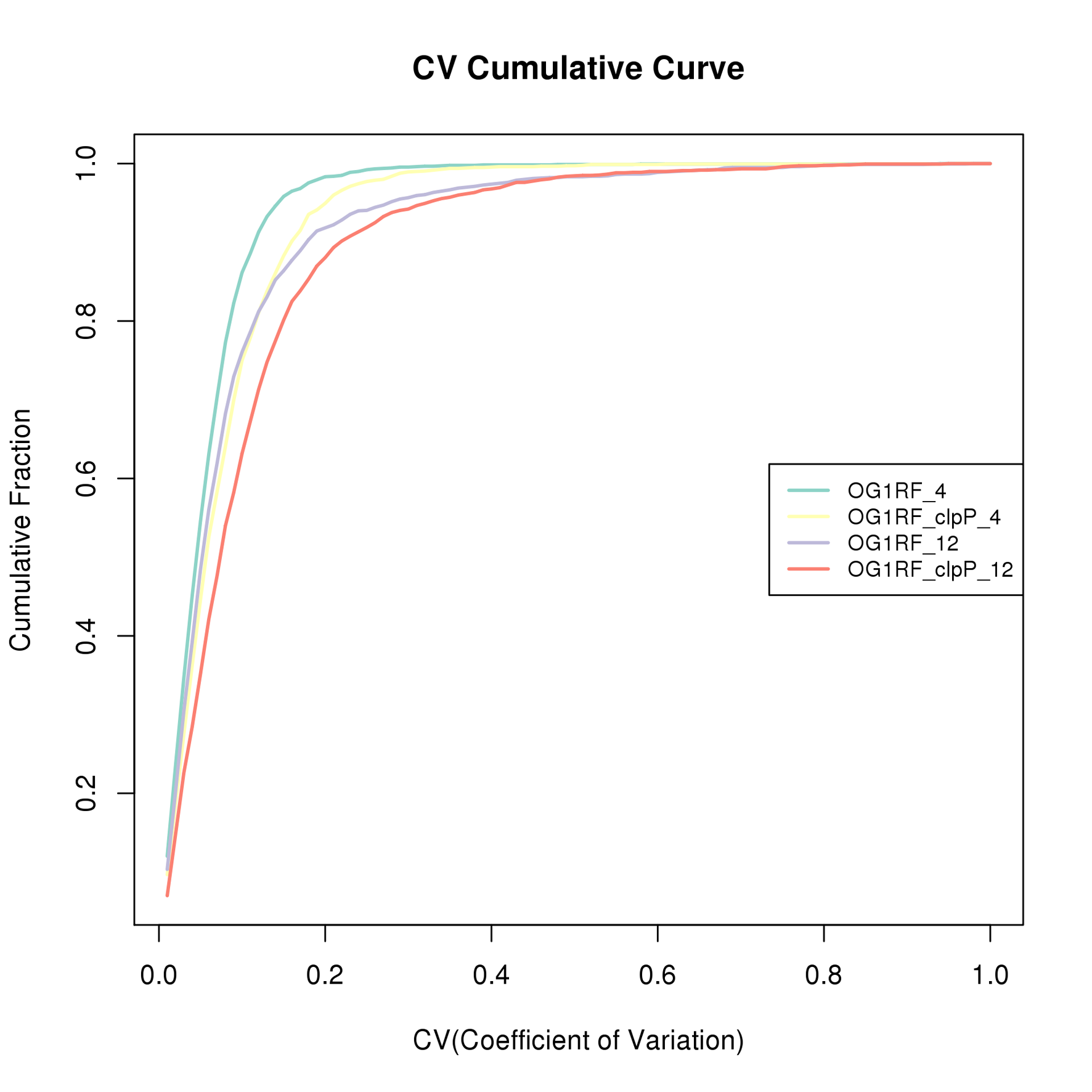

Supplement: Supplementary file 2 — Additional file 2: Figure S2. Coefficient of variation (CV) distributions for the two independent repetitions of the four group samples. The proteins were extracted from the E. faecalis OG1RF and its ΔclpP mutant strains and divided into four groups: strains cultured at 37 °C for 4 h to logarithmic phase were marked as OG1RF_4 or OG1RF_clpP_4; strains cultured at 37 °C for 12 h to stationary phase were marked as OG1RF_12 or OG1RF_clpP_12. The proteins extracted from each group included two independent biological repetitions, and the peptides were labeled with TMT6/10-plex reagents, then sequenced with the Orbitrap Q Exactive HF-X mass spectrometer. [file 12866_2020_1719_MOESM2_ESM.tif]

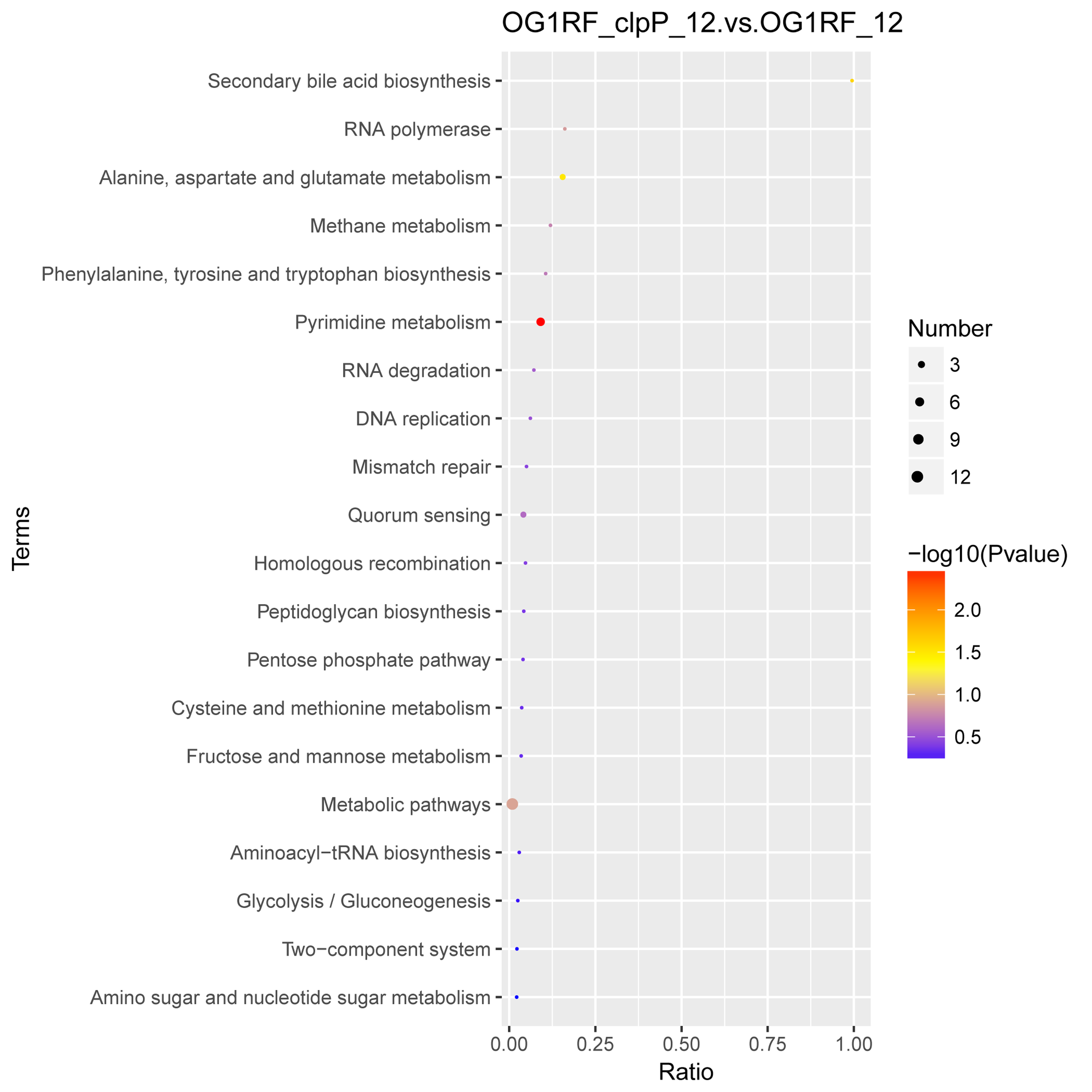

Supplement: Supplementary file 3 — Additional file 3: Figure S3. Kyoto Encyclopedia of Genes and Genomes (KEGG) analysis of differential abundance proteins (DAPs) (stationary phase). The protein family and pathway were analyzed using the KEGG database. [file 12866_2020_1719_MOESM3_ESM.tif]
