# Supplementary material for: ClpP participates in stress tolerance, biofilm formation, antimicrobial tolerance, and virulence of Enterococcus faecalis
Source: BMC Microbiol. 2020 Feb 7;20:30. doi: 10.1186/s12866-020-1719-9 (PMC7006429; doi:10.1186/s12866-020-1719-9)
Supplement: Supplementary file 4 — Additional file 4: Table S1. Antimicrobial susceptibility of E.faecalis determined by conventional broth macrodilution method. Table S2. Primers used for the RT-qPCR for the detection of RNA levels of differential abundance proteins. [file 12866_2020_1719_MOESM4_ESM.pdf]

Table S1 Antimicrobial susceptibility of *E.faecalis* determined by conventional broth macrodilution method

**Antimicrobials**	**Minimum inhibitory concentration (mg/L)**			
	**OG1RF**	**△*clpP***	**△*clpP*/pIB166::*clpP***	**△*clpP*/ pIB166**
Ampicillin	2	2	2	2
Vancomycin	2	2	2	2
Linezolid	2	2	2	2
Daptomycin	4	4	4	4
Levofloxacin	1	1	1	1
Rifampin	128	128	128	128
Minocycline	1	1	1	1
Gentamicin	16	16	16	16

Table S2 Primers used for the RT-qPCR for the detection of RNA levels of differential abundance proteins

Primers	Sequences (5' →3')	Location (CP002621.1)	PCR product size (bp)
OG1RF_11529-F	GCCTGGATATGATTGTTC	1591964-1591981	160
OG1RF_11529-R	CGTTAGAAGCATTGGTAA	1592106-1592123	
OG1RF_11526-F	CATCGCCATATTGAACTT	1587615-1587632	142
OG1RF_11526-R	TACACCATTATCCAGAACT	1587738-1587756	
OG1RF_12531-F	TTCGTAATCACTTCTTCTTCATAC	2681827-2681850	144
OG1RF_12531-R	CGGCTATATTCGCATTGTC	2681952-2681970	
OG1RF_12530-F	GGTGAATAAGGTAAGTATAAGTTG	2681435-2681458	94
OG1RF_12530-R	CAGAAGACATCCGTGAAG	2681511-2681528	
OG1RF_12246-F	TATGCTGCTTACCAAGAA	2368619-2368636	171
OG1RF_12246-R	ATGTCCATCTAACCAAGG	2368772-2368789	
OG1RF_12047-F	CGAACATCTCTTGGTAAG	2163367-2163384	191
OG1RF_12047-R	ATTATCTCTACTCGCTCTAA	2163538-2163557	
OG1RF_11076-F	CTCAATGGCTGGTTATCGTTA	1118547-1118567	81
OG1RF_11076-R	CGGATTCTTCTAAGTTCGTTCT	1118606-1118627	
OG1RF_12006-F	TCCGATACCTAAGTCAACATT	2117850-2117870	91
OG1RF_12006-R	AGAAGAACCAATCCGTCAA	2117922-2117940	
OG1RF_10167-F	TGCCTCTGCCTTGGATAA	171420-171437	123
OG1RF_10167-R	CCACGGTCGAAGACTACT	171525-171542	
OG1RF_10152-F	TTGGTAGCAGTAGAAGGTT	164816-164834	175
OG1RF_10152-R	CGTTATCAGAAGTTACTACAGAA	164968-164990	
OG1RF_12487-F	CATTGAGTGACGGTAGTA	2623318-2623335	158
OG1RF_12487-R	GTCTATCAGCAGTTGTTG	2623458-2623475	
OG1RF_10643-F	ATGAACTCTTATTACTATGC	680865-680884	152
OG1RF_10643-R	ATATCAATCTCTGCCAAT	680999-681016	
OG1RF_10009-F	CGACTATAAAGATACTGAAT	10363-10382	152
OG1RF_10009-R	TAACGAATGGTAATAATCC	10496-10514	
OG1RF_12468-F	CTCATCCAAGTCGCTTAA	2603594-2603611	89
OG1RF_12468-R	GAAACGAATCCGAGACAT	2603665-2603682	
OG1RF_12080-F	TTCAAGCCTAAGCCAGTT	2202715-2202732	107
OG1RF_12080-R	GGAGCAGCAGAAGAACAA	2202804-2202821	
OG1RF_12046-F	ACTGCTTCACTACCTTGGA	2162803-2162821	127
OG1RF_12046-R	AAGACCTGGCGGATAGAG	2162912-2162929	
OG1RF_11423-F	CACAGCAGGATAACTCAT	1483761-1483778	114
OG1RF_11423-R	TTATTAGATATTGAAGCGGTATT	1483852-1483874	
OG1RF_11424-F	AGCATAATGAATGACACT	1484179-1484196	149
OG1RF_11424-R	CTAAGGAAGGTCTAATCAA	1484309-1484327	
OG1RF_11429-F	CTAATGCTCCGTCAACAA	1490733-1490750	82
OG1RF_11429-R	GAATCAATGGCTGTCAATAC	1490795-1490814	
